# Pharmacometrics in the Age of Large Language Models: A Vision of the Future

**DOI:** 10.3390/pharmaceutics17101274

**Published:** 2025-09-29

**Authors:** Elena Maria Tosca, Ludovica Aiello, Alessandro De Carlo, Paolo Magni

**Affiliations:** Dipartimento di Ingegneria Industriale e dell’Informazione, Università degli Studi di Pavia, 27100 Pavia, Italy; elenamaria.tosca@unipv.it (E.M.T.); ludovica.aiello01@universitadipavia.it (L.A.); alessandro.decarlo01@universitadipavia.it (A.D.C.)

**Keywords:** large language models, ChatGPT, pharmacometrics, model-informed drug development

## Abstract

**Background**: Large Language Models (LLMs) have driven significant advances in artificial intelligence (AI), with transformative applications across numerous scientific fields, including biomedical research and drug development. However, despite growing interest in adjacent domains, their adoption in pharmacometrics, a discipline central to model-informed drug development (MIDD), remains limited. This study aims to systematically explore the potential role of LLMs across the pharmacometrics workflow, from data processing to model development and reporting. **Methods**: We conducted a comprehensive literature review to identify documented applications of LLMs in pharmacometrics. We also analyzed relevant use cases from related scientific domains and structured these insights into a conceptual framework outlining potential pharmacometrics tasks that could benefit from LLMs. **Results:** Our analysis revealed that studies reporting LLMs in pharmacometrics are few and mainly limited to code generation in general-purpose programming languages. Nonetheless, broader applications are theoretically plausible and technically feasible, including information retrieval and synthesis, data collection and formatting, model coding, PK/PD model development, support to PBPK and QSP modeling, report writing and pharmacometrics education. We also discussed visionary applications such as LLM-enabled predictive modeling and digital twins. However, challenges such as hallucinations, lack of reproducibility, and the underrepresentation of pharmacometrics data in training corpora limit the actual applicability. **Conclusions**: LLMs are unlikely to replace mechanistic pharmacometrics models but hold great potential as assistive tools. Realizing this potential will require domain-specific fine-tuning, retrieval-augmented strategies, and rigorous validation. A hybrid future, integrating human expertise, traditional modeling, and AI, could define the next frontier for innovation in MIDD.

## 1. Introduction

The emergence of Large Language Models (LLMs) marks a transformative milestone in artificial intelligence (AI). Built upon transformer-based architectures [1], these models have reshaped human–computer interaction through multiple applications such as virtual assistants, automated writing tools and real-time translation systems. They have also enabled groundbreaking applications across a wide spectrum of scientific disciplines, redefining the boundaries of what AI can accomplish in research and innovation.

LLMs are a class of deep learning models trained on vast textual corpora, including web content, the scientific literature and programming code. These models can perform a broad range of natural language processing (NLP) tasks, such as text generation, summarization, question answering, code synthesis, and contextual reasoning. Many LLMs also exhibit emergent behaviors, such as zero-shot and few-shot learning, enabling them to solve complex tasks with minimal or no task-specific training.

In healthcare and the life sciences, LLMs have already demonstrated substantial utility for clinical decision support [2], genetic consultancy [3] and drug discovery and development [4,5,6,7,8,9], including applications in de novo molecule design, ADMET property prediction, and patient-trial matching. These early successes highlight the disruptive potential of LLMs in transforming data-rich biomedical workflows.

Despite this momentum, the integration of LLMs into pharmacometrics remains limited and largely underexplored [10,11,12,13,14]. Pharmacometrics is a quantitative discipline that leverages mathematical modeling and simulation (M&S) to describe and predict pharmacokinetics (PK), pharmacodynamics (PD), and disease progression in response to therapeutic interventions. Aligned with the Model-informed Drug Development (MIDD) paradigm [15,16,17], pharmacometrics analyses inform evidence-based decision-making across all stages of drug development. Applications include supporting the translation of efficacy and safety findings from animals to humans [18,19,20], guiding dose escalation in first-in-human studies, optimizing study design or dose regimens in clinical trials, anticipating long-term study outcomes [21], helping characterize inter-patient variability, identifying influential covariates [22,23], guiding dosing strategy for special population and generating evidence for regulatory submissions. These analyses rely on a wide range of model approaches, such as Population PK/PD models, Physiologically Based-Pharmacokinetic (PBPK) models, Quantitative-System-Pharmacology (QSP) models and Time-To-Event (TTE), and involve complex and multidisciplinary workflows. Typical steps include contextualization of the pharmacological or clinical problem, data collection and formatting, model coding and implementation in specialized software (e.g., NONMEM, Monolix, or Stan), model identification and diagnostics, covariate analysis, simulation, and report writing. All of them require a combination of domain knowledge, quantitative skills, and iterative problem-solving. LLMs could assist in many of these tasks, from automation of repetitive steps, facilitation of technical writing, support in data manipulation or coding, and enhancement of knowledge synthesis. Nonetheless, this potential remains largely untapped in the pharmacometrics community.

This paper seeks to address a fundamental and timely question: how can LLMs be effectively leveraged across the pharmacometrics workflow to support MIDD, streamline routine tasks, and enhance the generation, interpretation, and communication of quantitative models? Therefore, unlike previous reviews that have either broadly discussed the integration of LLMs into the drug discovery and development process [4,5,6,7,8,9] or considered the application of traditional AI/ML methods in pharmacometrics [24,25,26], our perspective specifically centers on the intersection between LLMs and pharmacometric modeling. This targeted focus enables a more granular and application-oriented analysis of how LLMs may support modeling and simulation (M&S) activities within this highly specialized field.

In the following, we first introduce the main types and capabilities of LLMs. We then review the current, albeit extremely limited, literature on LLM applications in pharmacometrics. By reviewing recent LLMs results in related scientific fields, we provide a list of pharmacometrics tasks where these models could provide meaningful contributions. Finally, we discuss the potential, limitations, and ethical implications of deploying LLMs in pharmacometrics research and practice, aiming to outline a forward-looking vision for this emerging intersection.

## 2. Background on LLMs

LLMs are advanced AI systems designed to process, understand, and generate human-like text. They are based on deep neural network architectures known as transformers and introduced by Vaswani et al. in 2017 [1], which enables efficient modeling of long-range dependencies and contextual relationships within text sequences. Thanks to this architecture, LLMs have become the cornerstone of modern NLP, enabling sophisticated capabilities across a wide range of linguistic and reasoning tasks.

At the core of LLM functionalities lies the concept of token, a unit of text that typically represents a word, subword, or symbol. LLMs are trained to predict the most probable next token in a sequence, or to fill the missing ones in a incomplete sequence, given the surrounding context. This predictive ability is acquired through pretraining, on massive unlabeled corpora drawn from diverse sources. Training relies on self-supervised learning, in which the model generates learning signals by reconstructing masked or future parts of the input sequence. Through this mechanism, LLMs learn rich statistical and semantic representations of language, which they can later apply to downstream tasks.

Interaction with LLMs typically occurs through a prompt, i.e., an input string formulated by the user that may include questions, instructions, context, or examples. The model processes this prompt and generates an output token-by-token, leveraging the patterns and associations it learned during training. The design, clarity, and specificity of the prompt heavily influence the quality, relevance, and interpretability of the model output, a concept that underlies the emerging field of prompt engineering [27,28].

While LLMs share a common architectural foundation, they differ significantly in architecture types, scale (i.e., number of parameters), training strategies, objectives, and intended use. These distinctions have led to the emergence of distinct LLMs families, such as GPT, BERT, T5, and others, each with unique design choices and capabilities (Section 2.1). Furthermore, LLMs have demonstrated a range of emergent abilities [29], such as few-shot reasoning and in-context learning, which become more pronounced as model size increases (Section 2.2). Finally, LLMs can be broadly categorized based on their purpose and training data into general-purpose, domain-specific, or fine-tuned specialized models (Section 2.3).

### 2.1. Families of LLMs

Following the introduction of the transformer architecture by Vaswani et al. in 2017 [1], a wide range of LLM families have been proposed (see Table 1). These models differ in several key features, including transformer architecture configuration. They take one of the three main forms:

- *Encoder-only* models process input sequences bidirectionally, capturing contextual information from both left and right of each token. They are well-suited for classification, sentence similarity, and named entity recognition, but not for text generation, as they do not generate tokens autoregressively.
- *Decoder-only* are autoregressive models which are trained to predict the next token in a sequence given the previous tokens. This unidirectional approach makes them ideal for generative tasks such as text generation, dialog modeling, code completion, and open-ended question answering. These models underly most of the current generative LLMs.
- *Encoder–Decoder* models integrate both encoder and decoder blocks. This architecture allows the encoder to process the input text and pass contextualized representations to the decoder, which then generates the output sequence. Such models are particularly effective for machine translation, summarization, and structured question answering.

In addition to architecture, LLM families vary widely in scale, ranging from a few million to hundreds of billions of parameters, in training strategies and data, and intended application.

- The most widely known encoder-only LLM family is BERT (Bidirectional Encoder Representations from Transformers), originally developed by Google in 2018 [30] to advance natural language understanding tasks. Indeed, BERT is particularly effective for tasks such as information retrieval, text classification and aims to analyze and interpret text. Since the release of the original BERT model, its architecture has inspired the development of several derivative models by both Google and other research groups or companies (see Table 1).
- Among the decoder-only model series, one of the highly influential is GPT (Generative Pre-trained Transformer) introduced by OpenAI in 2018 [31]. GPT model, primarily designed for text generation, has subsequently evolved through several iterations (GPT-2 [32], GPT-3 [33], GPT-3.5 and GPT-4 [34]) that have substantially increased both model complexity and performance. In parallel, OpenAI also introduced InstructGPT [35] a fine-tuned version of GPT-3 optimized using reinforcement learning from human feedback (RLHF) [36]. InstructGPT was trained to follow user instructions more accurately and safely, forming the basis of ChatGPT [37], the popular conversational interface built on GPT models.

Another important decoder-only model family is LLaMA (Large Language Model Meta AI), developed by Meta [38]. The first version, LLaMA-1, was released in 2023 and have attracted the attention of the academic and research communities with open-access weights [39,40]. The subsequent LLaMA-2 model [41] improved performance and scalability and were optimized for instruction-following and alignment tasks. Most recently in 2024, LLaMA-3 announced in 2024, introduced enhanced reasoning and multilingual capabilities, positioning itself as a strong competitor to OpenAI GPT series.

Other emerging decoder-only model families include Claude, developed by Anthropic, and Gemini, developed by Google DeepMind, for which few information is publicly available. These models are designed to integrate advanced reasoning capabilities, security measures, and multimodal capabilities, expanding their applications beyond traditional NLP tasks.

Among the encoder–decoder LLM families, prominent groups include BART (Bidirectional and Auto-Regressive Transformers) developed by Facebook AI Research (FAIR) in 2019 [42], and T5 (Text-to-Text Transfer Transformer) introduced by Google in 2020 [43]. The encoder–decoder architecture makes them particularly effective in text-to-text transformation tasks, such as synthesis, paraphrasing, and translation. BART has been widely employed in content compression and text quality improvement, while T5 has been exploited in multi-task learning contexts, where a single model is adapted to perform multiple NLP tasks with high efficiency.

Models such as BERT, BART and T5 are open-source and freely available for research and development, while GPT, Claude and Gemini are proprietary and subject to commercial restrictions. LLaMA, while not completely open, has been made available for research under a noncommercial license, allowing limited use in academia and exploration.

**Table 1:** LLM families.

Family	Model	Developer	Year of Release	Number of Parameters	Pre-Training Corpora	Architecture
**BERT**	BERT (Base/Large) [30]	Google	2018	110M/340M	BookCorpus, Wikipedia	Encoder -only
	DistilBERT [44]	Hugging Face	2019	66B	BookCorpus, Wikipedia	
	RoBERTa (Base/Large) [45]	FAIR	2019	125M/355M	BookCorpus, CC-News, OpenWebText, Stories	
	AlBERT (Base/Large) [46]	Google	2019	12M/18M	BookCorpus, Wikipedia	
	ModernBERT (Base/Large) [47]	Hugging Face	2024	149M/395M	Undisclosed—2 trillion tokens from web documents, code, scientific articles, etc.	
	NeoBERT [48]	ByteDance AI Lab	2025	250M	RefinedWeb	
**GPT**	GPT-1 [31]	OpenAI	2018	117M	BookCorpus	Decoder -only
	GPT-2 [32]		2019	1.5B	BookCorpus, WebText	
	GPT-3 [33]		2020	175B	CommonCrawl, WebText, Wikipedia, Books1, Books2	
	GPT-3.5		2022	175B	Undisclosed	
	GPT-4 [34]		2023	Undisclosed	Undisclosed	
	GPT-4.5		2025	Undisclosed	Undisclosed	
**BART**	BART (Base/Large) [42]	FAIR	2019	140M/400M	BookCorpus, CC-News, OpenWebText, Stories	Encoder- Decoder
	mBART [49]		2020	610M	Common Crawl 25 languages subset (C25)	
**T5**	T5 [43]	Google	2020	60M-11B	Colossal Clean Crawled Corpus (C4)	Encoder- Decoder
	mT5 [50]		2021	13B	Multilingual Colossal Clean Crawled Corpus (101 languages) (mC4)	
	UL2 [51]		2022	20B	Colossal Clean Crawled Corpus (C4), other datasets	
**Llama**	LLaMa-1 [38]	Meta AI	2023	6.7M/13B/ 32.5B/65.2B	Common Crawl, C4, GitHub, Gutenberg Books3, Wikipedia, ArXiv, Stack Exchange	Decoder- only
	LLaMa-2 [41]		2023	7M/13B/ 34B/70B	2T tokens of curated data	
	LLaMa-3		2024	8B/70b	15T tokens; curated high-quality web, academic, code and multilingual corpora	

### 2.2. Emergent Abilities of LLMs

One of the most intriguing aspects of LLMs is their ability to exhibit emergent capabilities, behaviors that were not explicitly programmed or directly observed in smaller models, but which arise as the sizes of both model and training data increase. These emergent behaviors enable LLMs to generalize and reason in ways that go beyond the data they were trained on. Key examples include the following:

- *In-context learning:* the ability of an LLM to perform a new task by conditioning on information provided in the prompt at the inference time without the need of updating model parameters or additional re-training [33].
- *Few-shot or zero-shot learning*: the capacity of the model to generalize to unseen tasks either without any examples (zero-shot) or with only a few illustrative examples (few-shot) provided within the prompt [33].
- *Chain-of-thought reasoning*: the ability to generate intermediate reasoning steps that lead to a final answer, improving performance on complex tasks that require multi-step logical inference, mathematical reasoning, or structured problem-solving [52].

These emergent properties significantly enhance the versatility of LLMs and contribute to their growing applicability in scientific and technical domains, including pharmacometrics.

### 2.3. Classification of LLMs: General-Purpose, Purpose-Built, and Specialized Models

LLMs can be broadly categorized into three main types based on their training data and intended application scope.

- *General-Purpose LLMs:* These models are pre-trained on broad, diverse corpora including internet-scale text, code, news, encyclopedias, and books. Their goal is to acquire general linguistic and reasoning skills applicable across domains. They are not optimized for any specific task or field but exhibit strong performance across a wide range of NLP applications. For example, previously introduced GPT model series (OpenAI), Claude (Anthropic), and Gemini (Google DeepMind) are prominent general-purpose LLMs.
- *Purpose-Built LLMs:* These LLMs are trained from scratch exclusively or predominantly on domain-specific data (e.g., biomedical literature or clinical text). They are optimized from the start to understand the language, terminology, and context of a specific field. For example, BioGPT [53] and BioMedLM [54] are LLMs based on the GPT-2 architecture that were trained from scratch on a corpus of biomedical literature from PubMed, allowing to generate content and answer questions with higher relevance to biomedical research.
- *Specialized or Custom LLMs:* These are general-purpose LLMs that are subsequently fine-tuned with domain-specific data to improve performance in a targeted application area. Fine-tuning involves a retraining of the base model using curated datasets relevant to a specific task or domain. A well-known example is Codex, a derivative of GPT-3 fine-tuned on a vast corpus of programming code, enabling state-of-the-art performance in code generation, debugging, and language-to-code translation tasks [55]. Similarly, Med-PaLM [56], built on the PaLM architecture, was fine-tuned on medical question-answer datasets to improve performance on medical reasoning and diagnosis tasks. Examples from other domains include LegalBERT [57], a fine-tuned variant of BERT adapted for legal documents, and FinGPT [58], a model fine-tuned for financial analysis and reporting.

This classification is particularly relevant from relatively small research fields, such as pharmacometrics, where the choice between using a general-purpose assistant, a domain-specific model or a custom fine-tuned version can significantly impact accuracy, reliability, and applicability in modeling workflows.

## 3. Current Applications of LLMs in Pharmacometrics

This section critically reviews the existing literature on the use of LLMs in pharmacometrics. Although LLMs have been rapidly adopted across many biomedical and computational disciplines, their application within pharmacometrics remains underexplored. To date, only a handful of peer-reviewed articles or preprints have explicitly investigated the use of LLMs in the pharmacometrics workflow (see Table 2). These studies primarily focus on the use of general-purpose LLMs, such as ChatGPT, Copilot, Gemini and others, for assisting with code generation and model interpretation. The most frequently evaluated aspects are syntactic accuracy, task completion, reproducibility, and error handling. A notable exception is the recent work by Holt et al. [14], which investigated the LLMs capacity to support data-driven model building in dynamical pharmacological systems.

More in detail, Shin and Ramanathan investigated the performance of ChatGPT v4.0 across basic tasks relevant to PK analysis [11]. These included drafting the Introduction of a scientific manuscript to assess report writing ability, generating R code for graphical ex-ploration of PK data and non-compartment analysis, and solving a narrative PK problem. While ChatGPT performed adequately on tasks involving text writing or code generation and provided accurate information on principles and methods underlying PK data analysis, it exhibited several errors in numerical calculations. Furthermore, the output reproducibility across identical prompts was limited, leading to concerns about its reliability in regulated workflows. Simple prompt-engineering strategies were explored to improve output quality, leading to modest but insufficient improvements.

The generation of model code has been the central objective of most of the published studies. For instance, Cloesmeijer et al. [10] investigated the ChatGPT v3.5 ability to generate an R script for a PK model using the ‘desolve’ package. The model is a simple one-compartment model with inter-individual variability and allometric scaling of PK parameters. Then, the authors asked the LLM to simulate some PK scenarios and to develop a Shiny-based GUI to facilitate scenario exploration and output evaluation. Results suggested that ChatGPT could support simple PK modeling workflows and improve accessibility via interactive applications. However, occasional coding errors and task misinterpretation were reported. To mitigate these issues and enhance the reproducibility of ChatGPT outputs, Cloesmeijer et al. [10] recommended providing highly specific prompts, including package preferences and exact error messages, to guide the model toward correction and refinement.

Building on this, Herrero et al. [13] compared three LLMs (i.e., free versions of Mi-crosoft Copilot v4.0, ChatGPT v3.5 and Gemini 1.5 Pro (i.e., Bard v4.0)) across a more comprehensive pharmacometrics workflow. Tasks included implementing a two-compartment population PK model in R, estimating parameters using the ‘mapbayr’ package, and creating a Shiny-based dashboard for dosing simulations. They also evaluated the LLMs ability to generate diagnostic plots for model evaluation (e.g., VPC, GOF, distribution of random effects). While ChatGPT and Copilot outperformed Gemini in generating functional code for the explored PK workflows, all the LLMs demonstrated limitations in generalizability and complex diagnostics. Despite a complete explanation is challenging due to the inherent complexity of LLMs, the weaker performance of Gemini could be attributed to differences in training corpora and limited exposure to pharmaco-metrics programming resources.

Although the most widely adopted tools in pharmacometrics are NONMEM, Mono-lix, Stan and WinNonlin, previous studies focus exclusively on R programming language. Differently, Shin et al. explored the use of ChatGPT v3.5 and Gemini Ultra 1.0 to generate and interpret NONMEM control streams [12]. Investigated tasks involved designing a learning curriculum for NONMEM, outlining the typical structure of NONMEM control stream and generating codes for two different compartment PK models. Both LLMs demonstrated a basic understanding of NONMEM syntax and structure, with ChatGPT producing more complete and technically accurate outputs. However, both LLMs produced codes containing syntax or logical errors. This study assessed the LLMs debugging capabilities by feeding them the error messages generated by NONMEM. Results showed that, despite LLMs could suggest plausible corrections, their ability to autonomously resolve syntax issues remained limited.

More recently, Cha et al. [59] compared the performance of four LLMs (GPT-4o, Claude 3.5, Gemini 1.5 Pro, and LLaMa 3.2) in managing NONMEM output files. Specifically, LLMs were prompted to extract key information from NONMEM output streams and to generate the following: (1) structural model diagrams, (2) parameter summary tables and (3) analysis reports. Claude achieved the highest success and accuracy rates in generating structural model diagrams, followed by GPT-4o and Gemini, whereas LLaMa performed the worst across all evaluated models. For parameter extraction, GPT-4o successfully identified all parameters in both tested models, with Claude close behind at 90% accuracy. LLaMa again showed the lowest performance in this task. Additionally, GPT-4o was used to generate Python scripts to simulate two PK models of increasing complexity based on NONMEM outputs. While the model-specific code generated for the simpler scenario was executable and yielded plausible results, performance dropped with increased model complexity, revealing the current limitations of LLMs in accurately handling sophisticated simulation tasks without additional guidance or validation. Zheng et al. [60] further extended this line of investigation by evaluating seven OpenAI-based agents across 13 pharmacometric modeling tasks requiring NONMEM code generation, including compartmental PK models, direct and indirect response PD models, and complex target-mediated drug disposition models. Based on standardized criteria for evaluation, o1 and GPT-4.1 models were capable of generating highly accurate NONMEM code when provided with optimized prompts. While simpler model structures were generally handled well, performance declined as model complexity increased, especially in scenarios involving multiple compartments or indirect mechanisms. These findings underscore the need for expert oversight and iterative refinement when using LLMs in real modeling projects. Notably, the study also highlighted the potential of LLMs as educational aids for training new users in NONMEM coding, provided that their limitations are clearly understood and appropriate safeguards are in place to prevent overreliance on unverified outputs.

Holt et al. [14] went beyond code assistance and explored the potential of LLMs to directly develop pharmacometrics models and generate hypothesis. The authors introduced the Data-Driven Discovery (D3) framework, an AI-driven approach based on GPT-4, to completely automate the model building process. The D3 framework iteratively proposes, refines, and validates dynamical systems models combining three LLMs, each performing a specific task. Specifically, the first LLM generates pharmacometrics models using Python as coding language. The second LLM evaluates the previously generated models and suggested possible refinements. Lastly, the third LLM provides insights into the inclusion of additional features to the model. Applied to a Warfarin PK dataset, D3 produced a structurally novel model which incorporated non-linear effects, saturation kinetics and new covariate relationship and exhibited good predictive accuracy compared to existing models. Interestingly, the proposed model was positively reviewed by clinical pharmacologists, thus highlighting the enthusiasm around these novel AI-driven techniques. However, the authors themselves acknowledge that the proposed D3 method requires further evaluation across different pharmacometrics modeling scenarios. Moreover, it would be interesting to expand this workflow to classical population modeling tools (e.g., NONMEM, Monolix) to combine the potentialities of non-linear mixed effect modeling with LLM-based automation.

## 4. What Can LLMs Do for Pharmacometricians?

As discussed in the previous section, the documented applications of LLMs in pharmacometrics remain scarce and confined to basic tasks such as code generation, often in general-purpose language like R rather than pharmacometrics-specific ones. However, the potential of LLMs to support and enhance the pharmacometrics workflow extends far beyond these initial use cases and remains largely untapped.

**Figure 1:**
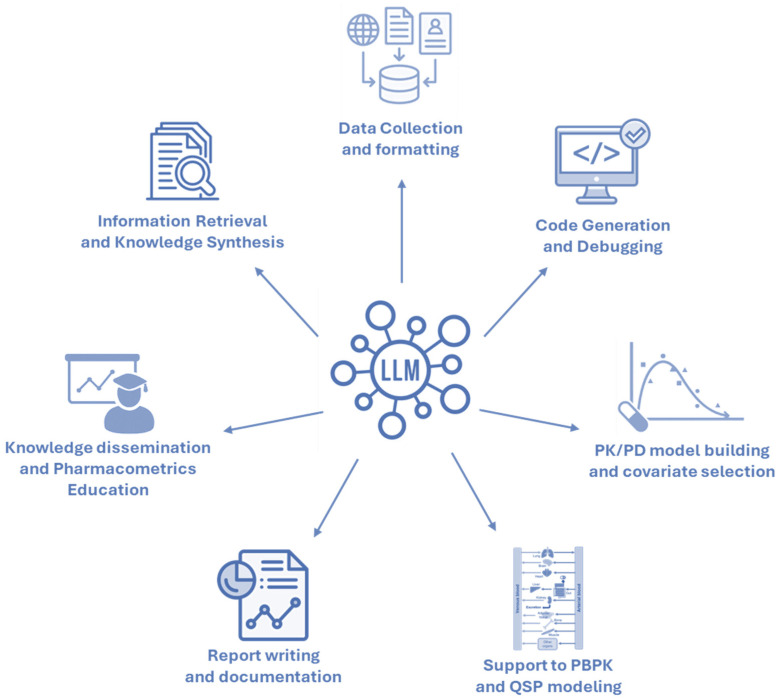
Possible applications of LLMs in pharmacometrics.

The objective of this section is to outline how LLMs could be strategically applied. To this end, we draw on lessons from the successful adoption of LLMs in adjacent scientific disciplines. This overview is not intended to be exhaustive. Rather, it aims to stimulate critical reflection and inspire pharmacometricians to consider how LLMs might be integrated into more advanced modeling, simulation, and decision-support workflows in both research and applied settings (see Figure 1).

### 4.1. Information Retrieval and Knowledge Synthesys

A comprehensive understanding of the clinical and pharmacological context is a critical first step in pharmacometrics modeling. This includes retrieving relevant information on disease mechanisms, biomarkers, drug characteristics, and prior modeling assumptions, which are essential to inform model structure, select covariates, and inform model parameterization. However, collecting and synthesizing this knowledge, often scattered across scientific publications, clinical guidelines, regulatory reports, and institutional documentation, can be highly time-consuming.

LLMs have demonstrated strong capabilities in information retrieval and knowledge synthesis, particularly within biomedical domains [61,62]. Domain-specific models such as BioGPT [53] and BioMedLM [54], trained from scratch on biomedical corpora using GPT-style architecture, were designed to support advanced NLP tasks including literature summarization. Even general-purpose LLMs have shown promising capabilities in biomedical information retrieval, even without domain-specific pretraining. For example, Gao et al. [63] demonstrated that ChatGPT, when properly prompted, could effectively verify drug–disease associations, achieving high accuracy in rejecting false claims and moderate accuracy in confirming true ones.

However, directly using LLMs as a search engine for information retrieval carries the risk of hallucinations [64], i.e., the generation of plausible but incorrect statements fictitious citations [65]. To mitigate this, a more reliable approach is the use of LLMs to summarize or interpret outputs from traditional information retrieval tools or curated databases. This hybrid strategy retains the efficiency and fluency of LLM-generated summaries while preserving scientific rigor through reference traceability.

In pharmacometrics, these capabilities could be strategically applied to automate or accelerate the initial knowledge synthesis phase of a modeling project. LLMs could assist in identifying relevant drug-disease or PK/PD relationships, extracting model structures or parameter values from prior studies, summarizing covariate effects, and generating structured representations of disease progression models. Moreover, they could support the drafting of the “model rationale” section of pharmacometrics reports by synthesizing current evidence, regulatory expectations, and prior modeling experience into coherent summaries (see Section 4.6). This would not only improve efficiency but also enhance transparency, reproducibility, and alignment with MIDD standards. Finally, a particularly promising direction is the application of LLMs to model-based meta-analysis (MBMA) [66,67]. By systematically extracting model structures, parameter estimates, and associated variances from published studies, LLMs could facilitate the automated aggregation of modeling evidence across compounds, indications, or populations. In addition, as also suggested by Lu et al. [7], LLMs could support MBMA tasks such as harmonizing heterogeneous study endpoints, standardizing terminology across datasets, and generating executable analysis code [67].

### 4.2. Data Collection and Formatting

Data collection and formatting are fundamental steps in pharmacometrics workflows, often requiring the integration of multiple and heterogeneous sources, including raw data from laboratory studies or clinical trials, observational studies, and, increasingly, real-world data (RWD) derived from electronic health records (EHRs) or clinical registries [68]. These data are often unstructured or semi-structured, vary in format and prone to inconsistencies and missing information, making the standardization process particularly labor-intensive.

Ensuring that such unstructured data are transformed into structured datasets suitable for modeling tools like NONMEM, Monolix or Stan is essential to ensure data usability and model reliability. For example, accurate temporal alignment of dosing events, biomarker measurements and response variables is critical in PK/PD modeling. Errors in this phase, such as misalignment or missing values, can bias parameter estimation and reduce model predictive performance. Given these complexities, this phase is time-consuming and demands meticulous attention from pharmacometricians.

A growing body of the literature suggests that LLMs offer novel solutions to streamline and enhance this complex process [7,9]. One promising application is the extraction and structuring of clinical data from EHRs and pathology reports. In the study conducted by Huang et al. [69], ChatGPT-3.5 was used to extract relevant information from over 1000 lung cancer pathology reports and 191 pediatric osteosarcoma reports in free text and convert them into structured data. Exploiting prompt engineering, the model achieved 89% classification accuracy, outperforming traditional NLP approaches and demonstrating the LLMs potential to automate RWD curation.

LLMs have also been explored in scientific literature mining. Rettenberger et al. [70] leveraged LLMs to automatically extract structured experimental information from dense biochemical publications, demonstrating the ability to distill key parameters from complex narratives. Additionally, Guiner-Miguelez et al. [71] used GPT-3.5 and FLAN-UL2 (belonging to the T5 family) to enrich metadata of ML datasets extracting key information from raw documentation. Across 12 scientific datasets, their tools, DataDoc Analyzer, achieved high accuracy and low hallucination rate in generate structured annotations, showing how LLMs can improve data documentation and compliance with emerging AI regulations.

In pharmacometrics, similar capabilities could streamline the collection and formatting of raw data, reduce formatting or transcription errors, and minimize manual workload, particularly when dealing with large-scale datasets. LLMs could also assist in the imputation of missing values, by inferring patterns from available data or referencing similar datasets, thereby contributing to data completeness and consistency. Finally, the ability of LLMs to extract structured information from unstructured clinical documents, such as EHRs, is of particular interest, given the increasing availability of RWD and the growing adoption of RWD-based modeling in pharmacometrics [68].

### 4.3. Code Generation and Debugging

As discussed in Section 3, code generation and debugging represent the most actively explored area for LLM application in pharmacometrics. Existing studies [10,11,13] have primarily investigated the ability of general-purpose LLMs (ChatGPT, Gemini and Microsoft Copilot) to assist in writing R scripts for PK modeling and simulation tasks. More recently, isolated attempts have also examined the LLMs capacity to generate NONMEM code from natural language prompts [12].

This trend mirrors broader developments in computer science, where LLMs are increasingly employed to support code development across various programming languages [72]. The relevance of this task led OpenAI to fine-tune GPT-3 on large repositories of open-source code from GitHub, resulting in Codex, an LLM capable of completing code or generating context-aware snippets based on natural language instructions [55]. Building on Codex, GitHub released Copilot [73], an AI-powered programming assistant that offers real-time suggestions for code lines or entire functions by drawing context from surrounding code and comments. The potential applications of LLMs in coding tasks are numerous and highly versatile: generating code from natural language instructions; providing templates or boilerplate code; debugging code, including interpreting log files, runtime errors or compiler messages; translating code across languages; annotating and refactoring code to improve readability, maintainability, and reproducibility; explaining code in plain language for educational or documentation purposes (see Section 4.5 and Section 4.6).

Such capabilities are highly relevant in pharmacometrics, where coding is central to model development, simulation, and evaluation workflows. However, the actual utility of LLMs in this context remains uncertain. Indeed, in the scientific literature, LLM-assisted code generation has focused mainly on widely used languages like Python, JavaScript, Java, C++, SQL, and R, which dominate both industrial software development and data science applications. Their prevalence in training corpora explains the strong performance of LLMs in these contexts. In contrast, pharmacometrics-specific languages, such as NONMEM control stream syntax or Monolix MLXTRAN, remain underrepresented in public corpora, potentially limiting the LLMs ability to generate syntactically correct and context-aware code for these tools.

To overcome this limitation, future research should explore fine-tuning LLMs on domain-specific code repositories, including annotated NONMEM scripts, Monolix projects, and R-based pharmacometric workflows. Such specialization could significantly enhance the performance and reliability of LLMs in pharmacometrics coding, paving the way for more automated and accessible model development.

### 4.4. PK/PD Model Building and Covariate Selection

Population PK/PD models, especially those based NLME approach, are central to pharmacometrics analysis. These models describe drug behavior and response variability across individuals, integrating both fixed effects (typical population parameters) and random effects (interindividual and residual variability). PK/PD model development is inherently iterative and concept-driven, requiring a combination of statistical modeling skills, pharmacological insight, and clinical understanding to define suitable model structures, test alternative hypotheses, and assess model adequacy. Key tasks include defining appropriate structural equations, specifying variability components, and justifying covariate effects on model parameters based on biological plausibility and data availability. Given its complexity, this stage represents one of the most intellectually demanding aspects of a pharmacometrics analysis.

Preliminary investigation by Holt et al. [14] suggests that LLMs can generate plausible model structures when prompted with system dynamics and descriptive input on drug-disease interactions. Although these findings remain exploratory, they highlight the potential of LLMs to support early model development.

Beyond suggesting initial PK/PD model structures, LLMs could assist with population model refinement by specifying interindividual variability terms, selecting covariate relationships or exploring residual error models. If fine-tuned on domain-specific content, LLMs might also contribute to interpretation of model diagnostics (GOF plots, visual predictive checks, shrinkage patterns, and other model evaluation metrics), potentially flagging inconsistencies or suggesting modifications. This would elevate LLMs from code assistant to decision-support tools during model development.

Regarding covariate screening and selection, LLMs could integrate quantitative information from clinical datasets with qualitative knowledge extracted from the literature. This hybrid reasoning capability might support the identification of mechanistically plausible covariate effects, enhance biological interpretability, and strengthen the rationale for covariate inclusion in the model.

### 4.5. Reshaping PBPK and QSP Modeling

PBPK and QSP models are among the most complex forms of modeling applied during drug development [74,75], offering comprehensive frameworks to simulate drug behavior within biological systems. They integrate heterogeneous data sources and involve the construction of multiscale mechanistic frameworks, facilitating predictions of drug ADME and PD effects across different species or patient subpopulations. Historically, these modeling paradigms have required extensive expert input, deep domain knowledge, and significant manual effort for model construction, parameterization, and validation.

Although still underexplored, LLMs hold significant potential to reshape these modeling paradigms as recently discussed by Androulakis et al. [76] and Goryanin et al. [77]. Their ability to process and synthesize vast bodies of biomedical literature could support hypothesis generation and facilitate the identification of relevant biological processes, disease pathways and mechanisms [4], drug–target or drug–drug interactions [78] to inform model structure and parameterization. Furthermore, due to their increasing capability of generating codes from natural language text (see Section 4.3), LLMs could translate narrative descriptions of physiological processes or drug mechanisms into modular code blocks or differential equations, facilitating the formalization of complex biological knowledge into simulation-ready models. This is particularly relevant given the equation-heavy nature of PBPK and QSP models, which often include dozens to hundreds of compartments and interconnections.

A key challenge in PBPK and QSP modeling is parameter calibration, which often requires retrieving physiological values, such as organ volumes or enzymatic rates, from the literature. LLMs can help automate this task (see Section 4.1), accelerating model setup and reducing manual effort. The work of Fatoki et al. [79] where ChatGPT was used to specify physiological parameter values of a PBPK model for oritavancin, represents a practical application of LLMs for this purpose.

Moreover, the potential of LLMs to interface with modeling platforms could support less-expert users in navigating complex models. Tools like Talk2Model [80] or Talk2BioModels [81] have demonstrated how LLM-powered agents can enable natural language interaction with models, promoting model interpretability and accessibility. Another example is provided by Kannan et al. [82], who systematically evaluated the capacity of several public LLMs to interact with and interpret formal biological models from repositories like BioModels, encoded in standardized formats including SBML, BioPAX, and SBGN. Their findings show that some LLMs can accurately summarize model structures, extract model parameters, propose perturbation experiments, and support simulation planning. These functionalities, although tested within the context of systems biology, are directly applicable to PBPK and QSP modeling, suggesting a path toward more transparent, interpretable, and interactive workflows in pharmacometrics.

### 4.6. Report Writing and Documentation

Structured reports of pharmacometrics analyses are fundamental during interactions with regulatory agencies in the MIDD era. These documents must ensure clarity and transparency, allowing diverse stakeholders, including pharmacometricians, clinicians, statisticians, and regulatory reviewers, to interpret findings and assess the relevance of the analysis. Reports must document all phases of the modeling workflow, from data preparation to model development, evaluation, simulation, and decision support, thereby facilitating reproducibility and regulatory review. Several regulatory guidelines [17,83,84] provide detailed recommendations on the appropriate structure and content of such reports, outlining the key components, best practices, and standards to ensure consistency and compliance with industry norms.

LLMs, with their advanced natural language generation capabilities, offer a transformative opportunity in this context. Although their application in pharmacometrics reporting has not yet been formally documented, their potential is significant. LLMs could be employed to convert a preliminary set of unstructured notes from an ongoing analysis into a well-organized report, consistent with regulatory requirements and reporting templates. They may also assist in translating code blocks, including model equations, estimation procedures or covariate analysis, etc., into natural language explanations. Furthermore, LLMs could be used to automatically manage outputs from commonly used software tools like NONMEM or Monolix, providing tables of parameter estimates, diagnostics, and simulation results to be included into structured textual summaries for reports or submissions. Emerging multimodal LLMs [85] further extend this potential by enabling interaction with non-textual content, including plots and tables, facilitating semi-automated report generation

Applications in related fields support this vision. In clinical settings, ChatGPT has been used to generate discharge summaries from clinical case notes [86]. In radiology, recent studies have evaluated the use of LLMs to convert unstructured reports into structured formats, improving interpretability and documentation efficiency [87,88,89,90,91,92,93]. These applications suggest that similar benefits could be realized in pharmacometrics, where structured reporting is critical for reproducibility and regulatory transparency.

### 4.7. Knowledge Dissemination and Pharmacometrics Education

Pharmacometrics requires multidisciplinary expertise in pharmacology, mathematics, statistics, and systems modeling, along with proficiency in dedicated software tools that often present steep learning curves [94,95]. Furthermore, model development processes typically involve high levels of human intervention and iterative refinement, while the interpretation of results demands a considerable degree of expertise and experience. As a result, there is an acknowledged shortage of qualified professionals to meet the growing workforce demands across industry, regulatory agencies, and academia.

LLMs are increasingly used in education and training [96], including medical [2], engineering [97], and computer science [98] fields. They can serve as virtual tutors, coding assistants, and interactive guides. These capabilities are especially valuable in fields requiring mastery of both conceptual knowledge and computational skills.

In pharmacometrics, LLMs could support learners by helping to write, interpret, and debug modeling code, acting as intelligent assistants capable of clarifying syntax, suggesting improvements, and identifying errors. Additionally, they may assist learners in navigating a complete model analysis workflow, from data preparation to structural model selection and covariate testing, providing dynamic, context-aware suggestions that emulate the guidance of an expert mentor. LLMs can also help explain model outputs in step-by-step form and translate technical scripts (e.g., NONMEM control streams) into accessible educational narratives.

Such tools could be particularly valuable for students or professionals with uneven skill sets, such as strong pharmacological training but limited programming experience. LLMs can adapt explanations to the user’s knowledge level and offer real-time clarification throughout the learning process. For example, Meyer et al. [99] demonstrated that ChatGPT enabled medical professionals with minimal programming experience to successfully develop a functioning laboratory application in R/Shiny, highlighting LLMs’ role in bridging digital skills gaps.

Moreover, LLMs have the potential to democratize pharmacometrics education, particularly in low-resource settings or regions lacking local expertise, thereby contributing to global capacity building in the field. Naturally, these benefits must be weighed against potential risks such as overreliance, propagation of incorrect information, and the importance of maintaining transparency in AI-assisted learning.

## 5. LLMs as Predictive Tools: Toward Pharmacometrics Model Replacement?

In the previous section, we discussed how LLMs can support various stages of the pharmacometrics workflow, ranging from information retrieval and knowledge synthesis to data preparation, model coding and development, documentation and educational activities. In these contexts, LLMs act primarily as assistive tools to streamline tasks typically performed by human modelers or domain-specific software.

This section shifts the focus to more speculative and transformative applications, where LLMs are envisioned as direct predictive engines, potentially challenging the role of conventional pharmacometrics models (i.e., PK/PD, PBPK or TTE models). Indeed, these approaches bypass the conventional pharmacometrics modeling by using LLMs to directly predict treatment response or clinical endpoints, such as survival.

A first promising application is in survival analysis [100], typically addressed in pharmacometrics using TTE models [101]. Hu et al. explored the use of GPT-based LLMs to predict post-operative survival and complications in lung cancer patients [102]. By prompting GPT-3.5 and GPT-4o-mini in a zero-shot setting, the authors predicted 1- to 5-year survival probabilities using structured clinical data from over 1200 patients, without any model-specific fine-tuning. GPT-4o-mini consistently outperformed standard logistic regression and achieved state-of-the-art performance across several endpoints. Similarly, NYUTron [103], a clinical-domain LLM trained on over 4 billion words from EHRs of 387,000 patients was fine-tuned to predict outcomes such in-hospital mortality. NYUTron demonstrated high accuracy and was prospectively deployed in a real-world clinical setting. While not designed explicitly to replace PK/PD or TTE models, its ability to predict clinical endpoints directly from unstructured notes illustrated the LLMs’ potential as high-performing, black-box alternatives.

Based on the LLM predicting capabilities, Derbal et al. proposed the OncoGPT framework, a conceptual architecture for predicting cancer treatment response [104,105]. OncoGPT proposed to learn mappings between therapeutic actions and patient outcomes by training on multimodal oncology data, including clinical, radiological, and molecular features. By tokenizing both treatment sequences and evolving disease states, the model would enable one-step-ahead predictions of treatment outcomes and be embedded within a closed-loop controller for adaptive therapy optimization. Although the theoretical feasibility, framework formalization (including the state-action space discretization) and training pipeline (comprising pretraining, fine-tuning and patient-specific personalization) were thoroughly outlined in the case study of metastatic castrate-sensitive prostate cancer [105], OncoGPT remains in the conceptual stage and has not yet been empirically implemented. Nevertheless, the proposal is promising and opens the door to further investigations. For instance, LLM-based predictors could to be integrated with reinforcement learning (RL), as previously performed for PK/PD models [26,106], to advance personalized, adaptive treatment strategies.

Another emerging research direction is the repurposing of LLMs for time series forecasting, relevant across multiple fields such as finance, climate modeling, traffic management and healthcare monitoring [107,108]. Transformer-based LLMs have been effectively adapted to process temporal data using a variety of strategies [108]. The simplest approach involves formatting time series as token sequences, encoding time stamps and numerical values into textual prompts that can be processed by the LLM similarly to natural language without any re-training or fine-tuning [109]. However, more advanced techniques have been investigated, including hybrid architectures that integrate traditional time-series encoders with LLM-based decoders [110,111]. In healthcare, Foresight [112], a GPT-2-based model trained on tokenized biomedical concepts extracted from EHRs, predicts future clinical events. Its extension, Foresight 2 [113], resulted from a fine-tuning of LLaMAv2-7B and Mistralv0.1-7B on larger and more diverse hospital datasets, improved temporal representations and prediction fidelity. In pharmacometrics, similar LLMs could, in principle, learn patient-specific concentration-time or response-time profiles directly from large-scale clinical or real-world data, without requiring explicit PK/PD modeling. However, the feasibility of LLMs as time-series forecasters remains debated. As highlighted by Tan et al. [114], multiple concerns persist about their robustness, generalization ability, and interpretability, which are essential attributes in the regulated context of drug development.

The potential forecasting capabilities of LLMs, combined with their strength in processing HER data, have also inspired their use in creating digital twins, i.e., virtual representations of patients used to simulate disease progression, evaluate treatment strategies and support precision medicine. For instance, the DT-GPT framework fine-tuned a biomedical LLMs, BioMistral 7B DARE [115], on multimodal hospital datasets to generate digital twins capable of modeling patient dynamics and forecasting future health states [116]. Similarly, Lammert et al. proposed a digital twin framework for precision oncology in patients with rare gynecological tumors, leveraging MedAlpaca, a biomedical-specific LLM [117]. Synthetic patients were constructed from real-world data and coded as narrative clinical histories, which were used as input to the LLM. Interesting, individualized treatment recommendations provided by the LLM closely resembled expert decision-making.

## 6. LLMs from Assistant to Collaborative Reasoning Partners: A Potential Revolution

In the previous sections, a variety of potential applications of LLMs within the pharmacometric workflow have been reviewed and proposed, ranging from information retrieval, data collection, code generation, covariate screening, and report writing, to more advanced uses such as hypothesis generators for physiologically based models and predictive tools. While these applications clearly demonstrate the value of LLMs in accelerating and facilitating multiple tasks that are otherwise time- and resource-intensive, when considered individually, they may give the impression that LLMs are just another layer of automation, on par with classical ML algorithms. However, such a view underestimates the truly transformative potential of LLMs. Two key features distinguish LLMs from traditional ML methods and position them as potentially revolutionary tools for the future of pharmacometrics.

First of all, LLMs are inherently generalist systems. Unlike most ML algorithms, which are typically trained and fine-tuned to perform one specific task, LLMs can handle a wide range of tasks, including reasoning, summarization, question answering, explanation, code generation, and translation, without requiring retraining. This flexibility stems from their pretraining on massive, diverse corpora and allows LLMs to seamlessly adapt to multiple contexts, even within highly specialized domains such as pharmacometrics.

Second, and arguably more importantly, LLMs possess emerging reasoning capabilities [29]. This allows them to operate not simply as passive tools performing a specific task, but as collaborative reasoning partners that can engage in natural language dialog, synthesize information across documents and timepoints, and support complex decision-making. As highlighted by Androulakis et al. [76], this represents a conceptual shift: transitioning LLMs from tools to active participants in scientific modeling, capable of integrating heterogeneous biomedical knowledge, generating hypotheses, proposing competing mechanistic explanations for observed PK/PD behaviors, and helping design simulation scenarios to test and refine model structures.

By engaging in interactive reasoning with users, rather than executing isolated tasks, LLMs could assist pharmacometricians in navigating ambiguous or uncertain model choices, interrogate consistency between clinical protocols and model assumptions, and trace the implications of parameter choices across simulation outputs. This collaborative mode opens the possibility for LLMs to enhance transparency, reproducibility, and decision-making within MIDD.

In this context, early-stage platforms, such as InsightRX Apollo AI [118], exemplify this collaborative vision. Apollo AI is an LLM-based software solution designed to assist in quantitative clinical pharmacology and translational sciences. With built-in human oversight, it integrates multiple LLM-based agents, each assigned to specific tasks, such as data cleaning and handling, conducting non-compartmental or population PK analyses, generating plots, and compiling model reports. These agents operate under the coordination of a central Planning Agent, which governs the sequence of steps required to complete the user-defined analysis. Although the platform is currently under development and its performance has not yet been documented in the scientific literature, it illustrates, at least conceptually, the potential of LLMs to accelerate and support pharmacometrics workflow, not merely as automation tools, but as orchestrators and collaborators in complex analytical pipelines.

## 7. Discussions

LLMs have revolutionized the field of AI, catalyzing unprecedented advancements across a wide range of scientific domains, including biomedical research and healthcare. However, despite this justified enthusiasm, it is essential to acknowledge that LLMs are not without limitations. Their application, particularly in high-stakes domains like healthcare, raises important concerns and challenges that must be addressed with caution [62,119]. These include risks of hallucination (i.e., generation of plausible but incorrect information), algorithmic bias, fairness issues, privacy breaches, and broader legal or ethical implications. Moreover, many LLMs lack comprehensive evaluations regarding their performance, robustness, and safety, raising legitimate concerns about their trustworthiness in critical applications. Addressing these limitations requires targeted mitigation strategies, including prompt engineering, external validation pipelines, audit trails, and alignment techniques. Additionally, frameworks such as retrieval-augmented generation (RAG) [120], which combine LLMs with external, curated knowledge bases, offer promising avenues to improve factual consistency and traceability. An additional strategy that has recently gained attention involves combining LLMs with other AI/ML techniques, such as supervised classifiers, neural networks, or Bayesian models, to create hybrid systems. These approaches have been shown to enhance LLM performance across key domains, language understanding, knowledge integration, and complex reasoning, especially in contexts characterized by limited training data [121,122].

In this perspective paper, we specifically reviewed and assessed the current and potential applications of LLMs in the field of pharmacometrics. Despite the transformative impact of LLMs in adjacent biomedical disciplines, our analysis reveals that their documented use in pharmacometrics remains extremely limited and largely restricted to supporting code generation in general-purpose languages such as R.

Yet, the potential for LLMs in pharmacometrics is far broader, as detailed in this work. From information retrieval and data structuring to model coding and development, documentation writing and education, LLMs could offer meaningful support for both routine and advanced tasks. Moreover, more speculative roles for LLMs, such as model-free prediction of treatment outcomes and integration into digital twin frameworks, can be envisioned by looking at how LLMs are already being employed in adjacent domains. While these task-specific applications clearly demonstrate the ability of LLMs to accelerate and facilitate key pharmacometric activities, they might also risk presenting LLMs as simple assistant tools, on par with other ML or automation strategies designed to increase efficiency. However, the true potential of LLMs is far more revolutionary. Unlike task-specific ML algorithms, LLMs are inherently versatile and capable of spanning across a wide range of modeling activities without retraining, thanks to their exposure to massive and diverse corpora. By leveraging their unique reasoning capacity, LLMs may evolve from supportive tools into collaborative reasoning partners, capable of synthesizing complex biomedical knowledge, supporting hypothesis generation, proposing mechanistic explanations, and guiding the iterative development and testing of pharmacometric models. These capabilities open new avenues for LLMs to actively participate in the scientific reasoning process behind pharmacometric modeling, potentially reshaping not only how models are built, but how modeling questions are framed and explored. Among the various domains within pharmacometrics, physiologically based models such as QSP arguably represent the most fertile ground for realizing this vision [76]. The multiscale and mechanism-rich nature of QSP models aligns well with the strengths of LLMs, which can integrate information from vast and heterogeneous biomedical sources, interpret complex model structures, and assist in formalizing biological knowledge into computational frameworks. Their ability to reason across textual, mathematical, and structural representations opens up new possibilities for how such models are conceptualized, explored, and communicated, potentially transforming QSP modeling into a more dynamic and collaborative scientific process.

In light of the transformative potential of LLMs, major pharmaceutical companies have expressed a growing interest in leveraging LLM-based tools for their workflows. As recently outlined in company blogs and press releases, several organizations, including Roche, Novartis, and AstraZeneca and others, are exploring internal applications of LLMs, such as domain-specific chat assistants. For example, Roche proposed PMx-AI Bot, a prototype assistant based on GPT-4 Turbo and trained on a combination of proprietary and public pharmacometrics resources which is designed to support tasks such as NONMEM code generation, covariate analysis, and simulation [123]. While such corporate interest is evident, its actual implementation, particularly in the context of pharmacometric workflows, remains entirely undocumented in the scientific literature.

To date, the real-world utility of LLMs in pharmacometrics remains to be rigorously demonstrated. Beyond the general limitations discussed earlier, the niche nature of pharmacometrics introduces additional challenges. Specifically, the underrepresentation of pharmacometrics data and examples in mainstream LLM training corpora may significantly limit their effectiveness and accuracy in this context. For example, while LLMs can generate high-quality code in well-represented languages like Python or R, their performance is likely to degrade in pharmacometrics-specific environments (such as NONMEM and Monolix) due to lack of exposure during pretraining. Fine-tuning LLMs on pharmacometrics-specific corpora could help address this gap. However, doing so presents its own challenges, such as the need to compile, curate, and annotate high-quality datasets, which are often proprietary and scattered across different software ecosystems. Community collaboration, transparency, and well-defined data governance protocols will be essential to enable such efforts. Alternatively, or in complement, RAG-based approaches represent a promising strategy. In RAG, the LLM is connected to an external database from which it retrieves task-relevant content at inference time. This setup allows pharmacometrics-specific resources to be dynamically accessed and cited without embedding all knowledge in the model parameters.

In summary, although LLMs have not yet revolutionized the field of pharmacometrics, their unprecedent reasoning capacity, synthesize diverse knowledge and engage in natural language dialog hold the promise to shift how pharmacometric models can be conceived, developed, and communicated. However, realizing this potential will require rigorous evaluation, domain-specific fine-tuning or retrieval strategies, and close attention to scientific and regulatory rigor. Looking ahead, the future of pharmacometrics in the age of LLMs will likely not be one of replacement, but of collaborative intelligence. A hybrid paradigm, combining human expertise, pharmacometrics modeling, and AI-driven tools, may define the next frontier of innovation within the MIDD framework.

With strategic investment and community-driven efforts, LLMs could evolve into indispensable allies, empowering pharmacometricians to meet the growing complexity and demands of modern drug development. Realizing this potential, however, will require continued methodological innovation, critical validation, and collaborative engagement across academia, industry, and regulatory bodies. Only through such collective commitment can LLMs be responsibly integrated into the pharmacometric toolbox and truly reshape the future of MIDD.

## Figures and Tables

**Table 2:** Summary of published applications of LLMs in Pharmacometrics.

Reference	Explored Pharmacometrics Task	Tested LLMs	Key Findings
Shin & Ramanathan (2024) [11]	Drafting an introduction for a PK analysis paper. Generating R code for PK data visualization and non-compartmental analysis. Solving a narrative PK problem.	ChatGPT-4	Good performance in text writing and code generation. Providing PK principles. Issue with numerical accuracy Limited reproducibility of outputs across identical prompts.
Cloesmeijer et al. (2023) [10]	Generating an R code for a one-compartment PK model. Simulating PK scenarios. Developing a Shiny-based GUI for scenario exploration and output evaluation.	ChatGPT-3.5	Ability to implement simple PK modeling workflows. Occasional coding errors and difficulty in understanding the task. Improvements with more detailed prompts.
Herrero et al. (2024) [13]	Generating an R code for a two-compartment PK model. Creating a Shiny-based dashboard for dosing simulations. Generating diagnostic plots.	ChatGPT-3.5 Gemini v4.0 Microsoft Copilot 4.0	Performance differences among LLMs. ChatGPT and Copilot produced more functional code. Challenges in task understanding and error correction.
Shin et al. (2024) [12]	Generating and interpreting NONMEM control streams. Creating a curriculum for learning NONMEM. Generating code for two-compartment PK models. Debugging NONMEM syntax errors.	ChatGPT-3.5 Gemini Ultra 1.0	Good understanding of NONMEM syntax. ChatGPT provided more complete and accurate outputs. Limited ability to resolve syntax issues autonomously.
Cha et al. (2025) [59]	Generating a structural model diagram from NONMEM output file. Generating parameter table from NONMEM output file. Generating analysis report from previous model diagram and parameter tables. Generating Python code for run simulation starting from NONMEM output files.	ChatGPT 4o Gemini 1.5 Pro Claude 3.5 Llama 3.2	Claude 3.5 and ChatGPT 4o obtained best success rate and accuracy in generating model diagram and parameter tables. Instead, Llama achieved the worst results. ChatGPT ability in converting NONMEM output in Python code strongly depends on model complexity.
Zheng et al. (2025) [60]	Generating NONMEM code for 13 models of different complexity, including compartment PK models, direct and indirect response PD models and Targeted Mediated Drug Disposition models.	GPT-4.1-mini GPT-4.1-nano GPT -4.1 GPT -4o-mini GPT -4o o1 o3-mini	o1 and GPT-4.1 demonstrated the best overall performance. Generation of an optimized prompt able to improve the accuracy of the LLM-generated NONMEM code.
Holt et al. (2024) [14]	Automating pharmacometrics model development and hypothesis generation. Iterative refinement and validation of PK models using three specialized LLMs.	GPT-4	Good ability in PK model generation. Necessary of further validation across different scenarios. Interest in expanding the workflow to integrate traditional modeling tools.

## Data Availability

No new data were created or analyzed in this study. Data sharing is not applicable to this article.

## Abbreviations

The following abbreviations are used in this manuscript:

ADMET	Absorption, Distribution, Metabolism, Elimination and Toxicity
AI	Artificial Intelligence
BART	Bidirectional Auto-Regressive Transformers
BERT	Bidirectional Encoder Representations from Transformers
EHR	Electronic Health Record
GPT	Generative pre-trained transformer
LLaMA	Large Language Model Meta AI
LLM	Large language Model
MBMA	Model-based meta-analysis
MIDD	Model-informed Drug Development
M&S	Modeling and Simulation
NLME	Non-linear Mixed Effect
NLP	Natural Language Processing
PBPK	Physiologically based pharmacokinetic
PK	Pharmacokinetics
PD	Pharmacodynamics
QSP	Quantitative System Pharmacology
RL	Reinforcement Learning
RLHF	Reinforcement Learning from Human Feeding
RWD	Real World Data
T5	Text-to-Text Transformer
TTE	Time-to-Event
